# Clinical parameter-based prediction of DNA methylation classification generates a prediction model of prognosis in patients with juvenile myelomonocytic leukemia

**DOI:** 10.1038/s41598-022-18733-4

**Published:** 2022-08-30

**Authors:** Takahiro Imaizumi, Julia Meyer, Manabu Wakamatsu, Hironobu Kitazawa, Norihiro Murakami, Yusuke Okuno, Taro Yoshida, Daichi Sajiki, Asahito Hama, Seiji Kojima, Yoshiyuki Takahashi, Mignon Loh, Elliot Stieglitz, Hideki Muramatsu

**Affiliations:** 1 Department of Advanced Medicine, Nagoya University Hospital, Nagoya, Japan; 2 Department of Pediatrics, Benioff Children’s Hospital, University of California, San Francisco, San Francisco, USA; 3 Department of Pediatrics, Nagoya University Graduate School of Medicine, 65 Tsurumai-cho, Showa-ku, Nagoya, Aichi 466-8560 Japan; 4 Department of Virology, Nagoya City University Graduate School of Medical Sciences, Nagoya, Japan; 5 Department of Hematology and Oncology, Children’s Medical Center, Japanese Red Cross Nagoya First Hospital, Nagoya, Japan

**Keywords:** Haematological cancer, Clinical genetics

## Abstract

Juvenile myelomonocytic leukemia (JMML) is a rare heterogeneous hematological malignancy of early childhood characterized by causative RAS pathway mutations. Classifying patients with JMML using global DNA methylation profiles is useful for risk stratification. We implemented machine learning algorithms (decision tree, support vector machine, and naïve Bayes) to produce a DNA methylation-based classification according to recent international consensus definitions using a well-characterized pooled cohort of patients with JMML (n = 128). DNA methylation was originally categorized into three subgroups: high methylation (HM), intermediate methylation (IM), and low methylation (LM), which is a trichotomized classification. We also dichotomized the subgroups as HM/IM and LM. The decision tree model showed high concordances with 450k-based methylation [82.3% (106/128) for the dichotomized and 83.6% (107/128) for the trichotomized subgroups, respectively]. With an independent cohort (n = 72), we confirmed that these models using both the dichotomized and trichotomized classifications were highly predictive of survival. Our study demonstrates that machine learning algorithms can generate clinical parameter-based models that predict the survival outcomes of patients with JMML and high accuracy. These models enabled us to rapidly and effectively identify candidates for augmented treatment following diagnosis.

## Introduction

Juvenile myelomonocytic leukemia (JMML) is a rare myelodysplastic/myeloproliferative neoplasm that occurs during infancy and early childhood. It is characterized by excessive myelomonocytic cell proliferation and granulocyte–macrophage colony-stimulating factor hypersensitivity^1–5^. More than 90% of patients with JMML harbor mutually exclusive somatic and/or germline mutations in canonical RAS pathway genes, including *PTPN11*, *NF1*, *NRAS*, *KRAS*, and *CBL*^6^*.*

Three independent studies described DNA methylation subgroups in JMML based on genome-wide DNA methylation data^7–9^. These studies demonstrated that high methylation subgroups were highly correlated with most of the established JMML risk factors, including older age^10,11^, higher hemoglobin F (HbF)^10,11^, lower platelet count^10,11^, *PTPN11*/*NF1* mutations^6,12^, the presence of secondary genetic events^13,14^, *LIN28B* overexpression^15^, and an AML-like expression profile^16^, and the high methylation subgroups were also associated with poor survival^17,18^. Recently, a joint analysis of the above three groups established an international consensus definition for the DNA methylation subgroups of JMML^19^.

Methylation subgrouping will be an important parameter for stratifying patients with JMML for treatment in upcoming clinical trials. However, the high cost and relatively long turnaround time will likely limit the clinical implementation of DNA methylation analysis to a few developed countries. Thus, the development of an inexpensive and rapid predictor of DNA methylation profiling is likely to benefit patients with JMML.

Decision trees, support vector machine (SVM), and naïve Bayes models are widely used supervised machine learning techniques. A decision tree is an intuitive approach of classification using the standard classification and a regression tree algorithm to select a split with the best optimization criterion, which is then recursively repeated for the two child nodes^20^. SVM classifies data by determining the linear decision boundary, also known as the hyperplane, that separates all data points of one class from another. The best hyperplane for SVM is the one with the largest margin between the two classes^21^. Naïve Bayes is a family of classifiers that implements Bayesian techniques to form a simple network based on previously established probabilities^22^.

In the present study, we performed a preliminary clinical parameter-based prediction of DNA methylation classification using a large cohort of patients with JMML via an international collaboration. We utilized the above-mentioned three machine learning algorithms to classify patients into two or three subgroups in accordance with DNA methylation profiles and assessed the clinical utility of these algorithms for predicting prognosis in an independent cohort. Implementation of these models will allow providers to stratify patients with JMML in accordance with recently published DNA methylation classifications.

## Results

### Training data

**Figure 1:**
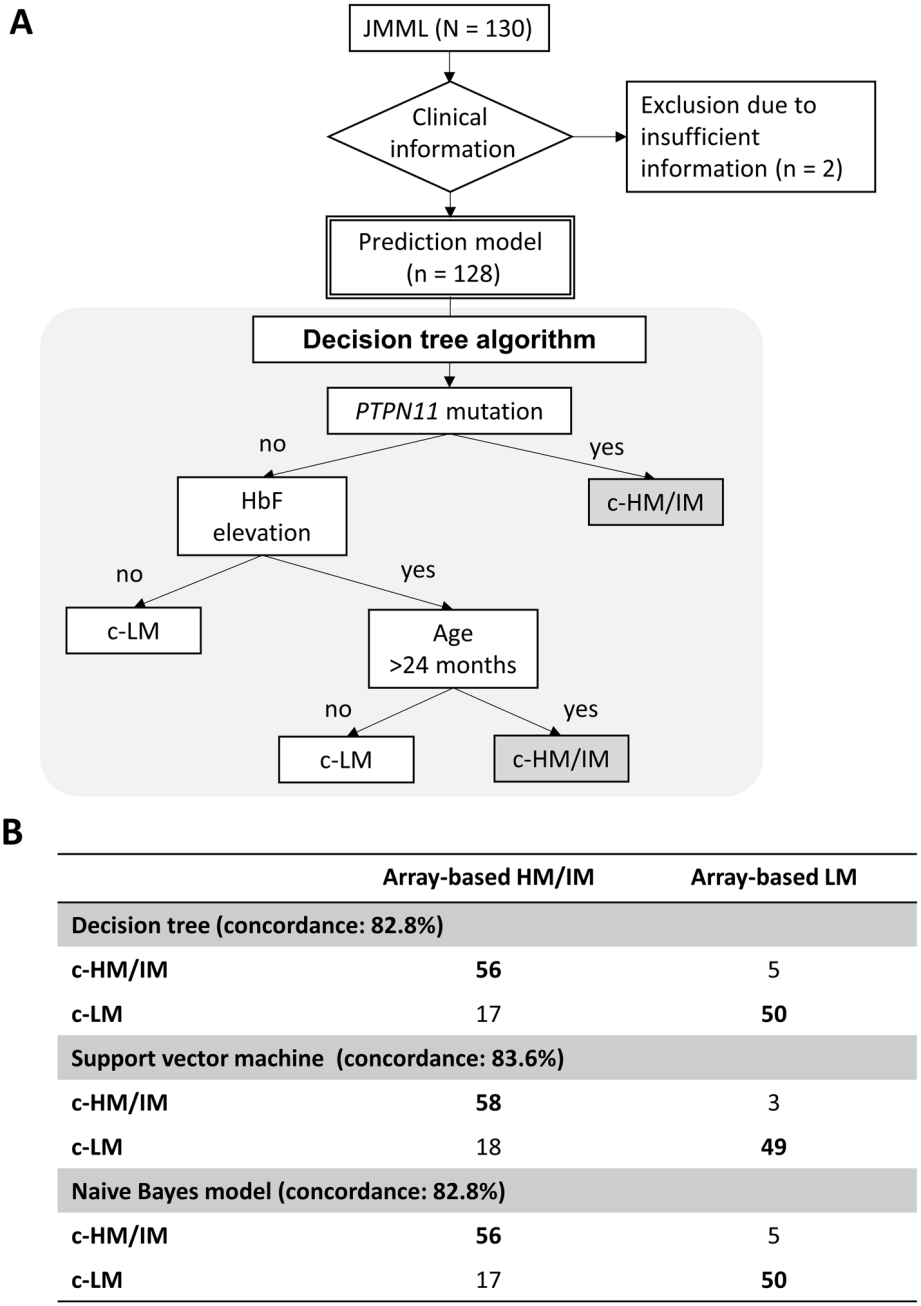
Clinical prediction model of dichotomized DNA methylation classification. (**A**) Flow diagram for DNA methylation profiles of patients used for the development of machine learning algorithms to predict DNA methylation classification. Patients from UCSF and the Japanese cohort who had methylation classification results were eligible for this analysis (N = 130). After excluding patients with incomplete clinical information (n = 2), 128 patients were included. Then, we implemented three machine learning algorithms. The figure shows a decision tree to classify into c-HM/IM and c-LM using widely available clinical information. (**B**) Three types of algorithms were implemented: decision tree, support vector machine, and naïve Bayes model. The concordance rates were 82.8%, 83.6%, and 82.8%, respectively. *JMML* juvenile myelomonocytic leukemia, *c-HM/IM* clinically predicted high/intermediate methylation, *c-LM* clinically predicted low methylation, *HM* high methylation, *IM* intermediate methylation, *LM* low methylation.

We collected previously published clinical data and 450k array DNA methylation data from 130 patients with JMML from two of our prior studies (93 from Nagoya University, Japan; 37 from University of California, San Francisco, USA)^8,9^. After excluding two patients with incomplete clinical information, we implemented three supervised machine learning algorithms, i.e., a decision tree, SVM, and a naïve Bayes model, to predict the dichotomized methylation profiles for the remaining 128 patients with JMML (Fig. 1A). The baseline characteristics of the aggregated cohort are summarized in Table 1. The concordance rate was 82.8% for the decision tree, 83.6% for the SVM, and 82.8% for the naïve Bayes model (Fig. 1B). The decision tree used age, HbF, and *PTPN11* mutation status to stratify patients. Based on the classification by the decision tree, we also compared the baseline characteristics between patients with clinically predicted LM (c-LM) and clinically predicted HM/IM (c-HM/IM). Univariable logistic regression models showed associations between clinical parameters and the dichotomized outcome (HM/IM vs. LM). The models indicated that the following factors were associated with the 450k array-based methylation classification: older age (≥ 24 months of age) (P = 6.7 × 10^−8^), age-adjusted HbF elevation (P = 8.2 × 10^−8^), the presence of *CBL* mutations (P = 6.8 × 10^−4^)*, PTPN11* mutations (P = 6.0 × 10^−8^), *NF1* mutations (P = 0.017), and *NRAS* mutations (P = 0.003). Lower platelet count and *KRAS* mutations were not significantly associated factors (P = 0.67 and 0.98, respectively) (Table 2).

**Table 1:** Baseline characteristics of the training and validation cohorts.

Covariates		Training	Validation	P value
Cohort, n (%)	UCSF	36 (28.1)	19 (26.4)	0.070
	Japan	92 (71.9)	53 (73.6)	
Age, n (%)	< 24 months	79 (61.7)	46 (63.9)	0.76
	≥ 24 months	49 (38.3)	26 (36.1)	
	Missing	0 (0)	0 (0)	
Sex	Female	86 (67.2)	33 (45.8)	0.003
	Male	42 (32.8)	39 (54.2)	
	Missing	0 (0)	0 (0)	
HbF	Normal	59 (46.1)	35 (48.6)	0.66
	Elevated	69 (53.9)	36 (50)	
	Missing	0 (0)	1 (1.4)	
Platelets	> 33 × 10^9^/L	66 (51.6)	60 (83.3)	< 0.001
	≤ 33 × 10^9^/L	61 (47.7)	12 (16.7)	
	Missing	1 (0.8)	0 (0)	
Monosomy 7	Positive	9 (7)	9 (12.5)	> 0.99
	Negative	119 (93)	61 (84.7)	
	Missing	0 (0)	2 (2.8)	
*CBL*	WT	106 (82.8)	63 (87.5)	0.38
	Mutated	22 (17.2)	9 (12.5)	
	Missing	0 (0)	0 (0)	
*PTPN11*	WT	82 (64.1)	50 (69.4)	0.44
	Mutated	46 (35.9)	22 (30.6)	
	Missing	0 (0)	0 (0)	
*NF1*	WT	114 (89.1)	70 (97.2)	0.041
	Mutated	14 (10.9)	2 (2.8)	
	Missing	0 (0)	0 (0)	
*NRAS*	WT	110 (85.9)	59 (81.9)	0.45
	Mutated	18 (14.1)	13 (18.1)	
	Missing	0 (0)	0 (0)	
*KRAS*	WT	109 (85.2)	57 (79.2)	0.28
	Mutated	19 (14.8)	15 (20.8)	
	Missing	0 (0)	0 (0)	
450k-based methylation	HM	48 (37.5)	NA	
	IM	19 (14.8)	NA	
	LM	61 (47.7)	NA	

*UCSF* University of California, San Francisco, *HbF* hemoglobin F, *WT* wild type, *HM* high methylation, *IM* intermediate methylation, *LM* low methylation, *NA* not available.

**Table 2:** Baseline characteristics between 450k-based HM/IM and LM and unadjusted logistic regression to predict 450k-based HM/IM as compared with 450k-based LM for each parameter in the training dataset.

Covariates		HM/IM (n = 67)	LM (n = 61)	OR (95% CI)	P value
Age	≥ 24 months, n (%)	42 (62.7)	7 (11.5)	13.0 (5.11–32.9)	6.7 × 10^−8^*
HbF	Elevated, n (%)	52 (77.6)	17 (27.9)	8.97 (4.02–20.0)	8.2 × 10^−8^*
Platelets	≤ 33 × 10^9^/L, n (%)	31 (46.3)	30 (50.0)	0.86 (0.43–1.73)	0.67
Monosomy 7	Positive, n (%)	9 (13.4)	0 (0)	12.8 (1.95–+ ∞)^†^	0.005*
*CBL*	Mutated, n (%)	1 (1.5)	21 (34.4)	0.029 (0.004–0.22)	6.8 × 10^−4^*
*PTPN11*	Mutated, n (%)	41 (61.2)	5 (8.2)	17.7 (6.25–49.9)	6.0 × 10^−8^*
*NF1*	Mutated, n (%)	12 (17.9)	2 (3.3)	6.44 (1.38–30.1)	0.017*
*NRAS*	Mutated, n (%)	3 (4.5)	15 (24.6)	0.14 (0.039–0.53)	0.003*
*KRAS*	Mutated, n (%)	10 (14.9)	9 (14.8)	1.01 (0.38–2.69)	0.98

*HM/IM* high/intermediate methylation, *LM* low methylation, *OR* odds ratio, *CI* confidence interval, *HbF* hemoglobin F, *WT* wild type.

*P < 0.05.

^†^Exact logistic regression was employed.

**Figure 2:**
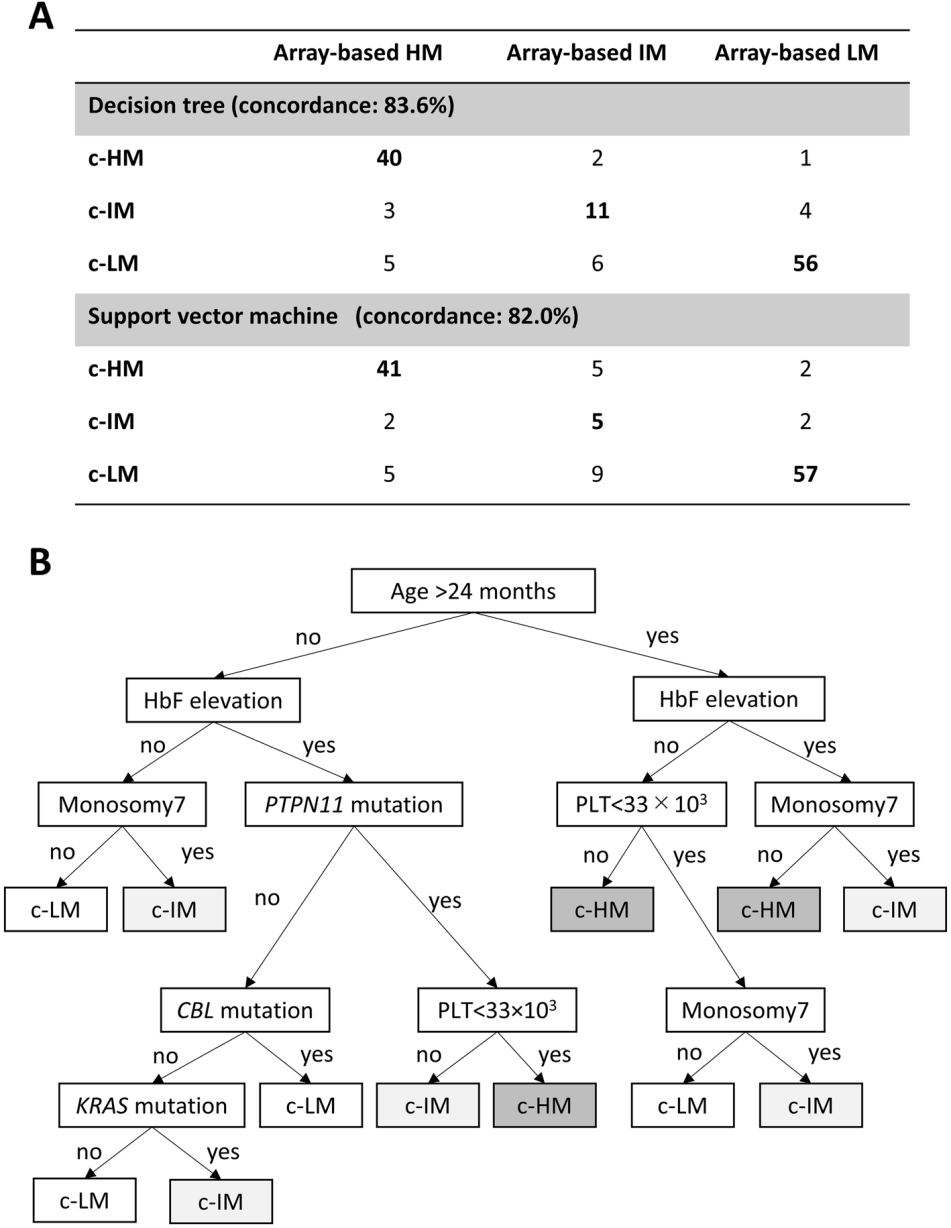
Clinical prediction model of trichotomized DNA methylation classification. (**A**) To predict DNA methylation classification, we attempted to implement three types of algorithms (decision tree, support vector machine, and naïve Bayes model). However, the naïve Bayes model failed in this analysis. The concordance rates were 83.6% for the decision tree and 82.0% for the support vector machine. (**B**) Decision tree to classify into c-HM, c-IM, and c-LM using the following clinical information: age, HbF, platelet counts, *PTPN11*, and *KRAS* mutation. *c-HM* clinically predicted high methylation, *c-IM* clinically predicted intermediate methylation, *c-LM* clinically predicted low methylation, *HM* high methylation, *IM* intermediate methylation, *LM* low methylation.

We also implemented the three algorithms to mimicking three DNA methylation subgroups. There was a high concordance rate between predicted and actual methylation classification profiles in the decision tree (82.8%) and the SVM (83.6%), although the naïve Bayes model failed to classify the patients into three subgroups (Fig. 2A). The decision tree in this analysis used the following variables: age, HbF, monosomy of chromosome 7 (monosomy 7), platelet counts, and *PTPN11, CBL, and KRAS* mutations (Fig. 2B).

### Survival analysis

In survival analyses, the incidence rate of death was 10.6 [95% confidence interval (CI) 7.9–14.2] per 100 patient-years and that of transplantation was 55.2 (95% CI 45.3–67.1) per 100 patient-years in the training cohort, while it was 10.0 (95% CI 6.1–16.3) and 111 (95% CI 86.1–143.4) respectively, in the validation cohort. The median transplantation-free survival (TFS) was 6.5 and 6.0 months in the training and validation cohorts, respectively.

**Figure 3:**
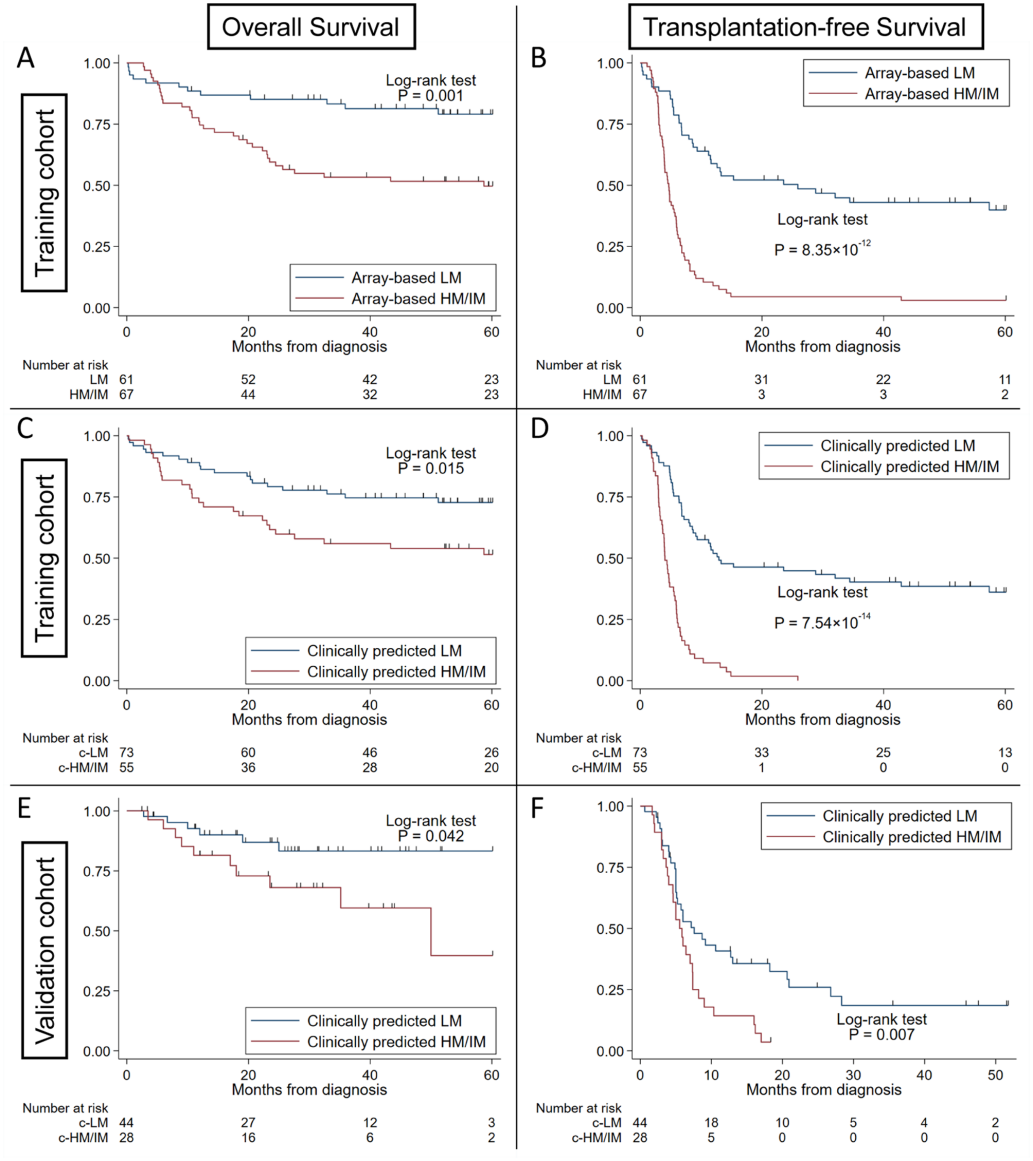
Overall survival and transplantation-free survival based on dichotomized DNA methylation classification. (**A**) Overall survival (OS) by array-based DNA methylation classification in the training cohort. (**B**) Transplantation-free survival (TFS) by array-based DNA methylation classification in the training cohort. (**C**) OS by clinically predicted methylation classification in the training cohort. (**D**) TFS by clinically predicted methylation classification in the training cohort. (**E**) OS in the validation cohort. (**F**) TFS in the validation cohort. Survival curves were estimated using the Kaplan–Meier method, and statistical tests were performed using the log-rank test. Array-based HM/IM or LM was significantly associated with both OS and TFS in the training cohort (P = 0.001 and 8.35 × 10^−12^). Clinically predicted HM/IM or LM was significantly associated with both OS and TFS in the training cohort (P = 0.015 and 7.54 × 10^−14^, respectively) and associated with both OS and TFS in the validation cohort (P = 0.042 and 0.007, respectively). *OS* overall survival, *TFS* transplantation-free survival.

Patients with array-based LM had significantly higher overall survival (OS) and TFS than those with array-based HM/IM (P = 0.001 and 8.35 × 10^−12^, respectively) (Fig. 3A,B). Based on the decision tree algorithm, patients with c-LM (n = 73) had higher OS and TFS than those with c-HM/IM (n = 55) (P = 0.015 and 7.54 × 10^−14^, respectively) (Fig. 3C,D). The SVM classified patients into a c-LM subgroup with 76 patients and a c-HM/IM subgroup with 52 patients; it showed a higher OS and TFS in the c-LM group (P = 0.004 and 7.15 × 10^−14^, respectively) (Supplementary Fig. S1A,B). The naïve Bayes model classified patients into a c-LM subgroup with 73 patients and a c-HM/IM subgroup with 55 patients, and showed a higher OS and TFS in the c-LM group (P = 0.015 and 7.54 × 10^−14^, respectively) (Supplementary Fig. S2A,B).

**Figure 4:**
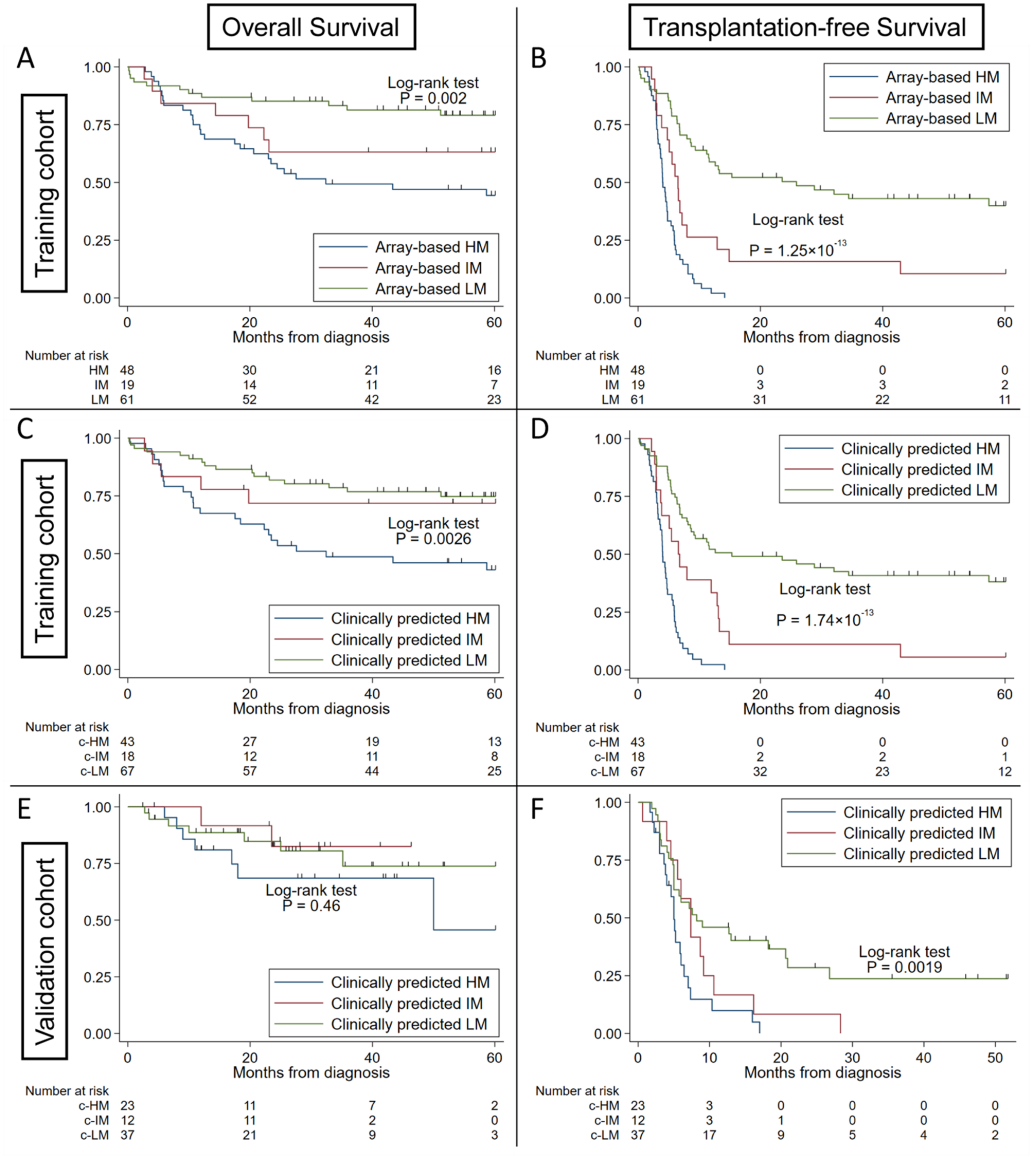
Overall survival and transplantation-free survival based on trichotomized DNA methylation subgroups (HM, IM, and LM). (**A**) Overall survival (OS) by array-based DNA methylation classification in the training cohort. (**B**) Transplantation-free survival (TFS) by array-based DNA methylation classification in the training cohort. (**C**) OS by clinically predicted methylation classification in the training cohort. (**D**) TFS by clinically predicted methylation classification in the training cohort. (**E**) OS in the validation cohort. (**F**) TFS in the validation cohort. Survival curves were estimated using the Kaplan–Meier method, and statistical tests were performed using the log-rank test. Array-based subgroups were significantly different in both OS and TFS in the training cohort (P = 0.002 and 1.25 × 10^−13^). Clinically predicted HM/IM or LM was significantly associated with both OS and TFS in the training cohort (P = 0.0026 and 1.74 × 10^−13^, respectively) and with TFS in the validation cohort (P = 0.0019). *OS* overall survival, *TFS* transplantation-free survival.

Next, we implemented these algorithms to analyze the survival of patients in these three methylation subgroups. Figure 4A,B show the Kaplan–Meier estimates comparing array-based HM, IM, and LM. Both OS and TFS were significantly different across the array-based methylation subgroups (P = 0.002 and 1.25 × 10^−13^, respectively). Regarding the preliminary clinically predicted methylation subgroups, both OS and TFS were significantly different across the subgroups in the decision tree (P = 0.0026 and 1.74 × 10^−13^, respectively) (Fig. 4C,D) and the SVM (P = 6.05 × 10^−5^ and 1.51 × 10^−13^, respectively) (Supplementary Fig. S3A,B). The naïve Bayes model failed to classify patients into three subgroups.

### Validation using an independent cohort

We applied the clinical parameter-based prediction models to an independent cohort (n = 72) (Table 1). Using dichotomized methylation classification, preliminary clinically predicted methylation classification showed a statistically significant difference between c-LM and c-HM/IM in both OS and TFS based on the decision tree algorithm (P = 0.042 and 0.007, respectively) (Fig. 3E,F), the SVM (P = 0.17 and 0.025, respectively) (Supplementary Fig. S1C,D), and the naïve Bayes model (P = 0.020 and 0.001, respectively) (Supplementary Fig. S2C,D). The clinically predicted trichotomized DNA methylation subgroup (cLM, c-IM, and c-HM) analyses showed a significant difference in TFS (P = 0.0019) but not in OS (P = 0.46) in the decision tree algorithm (Fig. 4E,F), as it did in the SVM (P = 0.0068 for TFS and 0.36 for OS, respectively) (Supplementary Fig. S3C,D).

### Comparison of prognostic performance by the models

The prognostic performance of each model was compared using Harrel’s C statistic (Table 3). Compared to array-based methylation classification, the C-statistics for the models derived from machine learning algorithms were comparable for both OS and TFS in the training cohort. In the validation cohort, the decision tree-based reclassification model was comparable to the other models for both OS and TFS.

**Table 3:** Comparison of C-statistics in the models.

Classification	C-statistics	∆C-statistics	P value
**Training cohort**			
**OS**			
Array-based	0.616 (0.542–0.690)	Reference	
Decision tree-based	0.588 (0.513–0.663)	− 0.028 (− 0.093 to 0.037)	0.39
SVM-based	0.603 (0.529–0.677)	− 0.013 (− 0.077 to 0.050)	0.68
Naïve Bayes-based	0.588 (0.513–0.663)	− 0.028 (− 0.093 to 0.037)	0.39
**TFS**			
Array-based	0.657 (0.610–0.705)	Reference	
Decision tree-based	0.663 (0.620–0.706)	0.006 (− 0.037 to 0.048)	0.79
SVM-based	0.659 (0.617–0.702)	0.002 (− 0.036 to 0.041)	0.92
Naïve Bayes-based	0.663 (0.620–0.706)	0.006 (− 0.037 to 0.048)	0.79
**Validation cohort**			
**OS**			
Decision tree-based	0.573 (0.440–0.705)	Reference	
SVM-based	0.624 (0.492–0.757)	0.052 (− 0.035 to 0.138)	0.24
Naïve Bayes-based	0.560 (0.426–0.693)	− 0.013 (− 0.104 to 0.078)	0.78
**TFS**			
Decision tree-based	0.553 (0.486–0.619)	Reference	
SVM-based	0.582 (0.516–0.649)	0.030 (− 0.014 to 0.074)	0.18
Naïve Bayes-based	0.586 (0.515–0.657)	0.034 (− 0.022 to 0.090)	0.23

C-statistics represent Harrel’s C concordance statistics.

*OS* overall survival, *TFS* transplantation-free survival.

## Discussion

In the present study, we implemented machine learning algorithms to develop preliminary clinical parameter-based prediction models of methylation profiles using an international cohort of patients with JMML and demonstrated a high concordance between DNA methylation and clinically predicted DNA methylation subgroups. We also assessed the validity of the models for assessing survival in an independent cohort. To our knowledge, this was the first study to develop clinical parameter-based models to predict an international consensus definition of DNA methylation subgroups in JMML^19^.

Notably, we developed prognostic models based on methylation classification, which may have the advantage of being able to predict high-risk JMML regardless of future changes in treatment, while the prognosis-based approach may not be applicable in future patients due to medical advances. In this regard, we believe methylation-based prediction would be a better approach to predict patients’ prognosis.

The models consisted of well-known clinical predictors, and the mechanisms for each parameter can be accounted for by prior knowledge^4,11^. In our clinical models, age, age-adjusted HbF elevation, and *PTPN11* mutation were specified as classifiers to dichotomize the methylation profile, while age, age-adjusted HbF elevation, platelet count, *PTPN11* and *CBL* mutations, and monosomy 7 were utilized as classification factors for the three methylation subgroups. Among the three machine learning algorithms we tested, namely, decision tree, SVM, and naïve Bayes models, decision tree algorithms were more intuitive than the others and were in line with our clinical knowledge as well as previous studies. Furthermore, the discrimination performance of the decision tree algorithm-based model was comparable to the array-based model in the training cohort and the other models in the validation cohort. Although the concordance between predicted and array-based classification was quite high for both dichotomous and trichotomous methylation profiles, dichotomized methylation enabled us to stratify patients with JMML simply and effectively for both OS and TFS.

Our models enabled us to effectively dichotomize patients with JMML (c-HM/IM and c-LM). In the training cohort, the 2-year TFS was nearly 50% in the c-LM group. The models were also capable of stratifying patients for augmented treatment for c-HM/IM patients. There is also clinical utility in identifying the patients most likely to survive in the absence of hematopoietic stem cell transplantation, in particular in developing countries.

The decision tree algorithm successfully stratified patients with JMML into three groups, as did the consensus classification in the previous study. However, the decision tree algorithm fitted to the validation cohort for categorization into three methylation groups showed a significant difference in TFS but not in OS. This likely reflects the shorter duration of follow-up in the validation cohort. Using a consensus classification, TFS in the HM and IM groups was equally poor in the European, American, and Japanese cohorts^19^. These findings suggest that the HM and IM groups may be biologically similar to one another.

There were some limitations in this study. First, the sample size in our study was relatively small. However, this international collaborative study contained a substantial number of patients with JMML despite the rarity of the disease, and machine learning algorithms enabled us to enhance the statistical power as well as the precision of prediction. Second, because of the lack of methylation profile data in the validation cohort, we have not been able to test whether the risk classifier established in this study can reproduce the methylation profile itself, and validation is needed in future studies.

In conclusion, we successfully developed preliminary clinical parameter-based DNA methylation prediction models and tested them in an independent cohort. These models stratified patients based on the necessity for more intensive treatment, including transplantation. With additional validation, these models will be helpful for patients with JMML worldwide.

## Methods

### Study design

We developed prediction models of methylation classification using machine learning algorithms in a training cohort and assessed the models’ survival analysis efficacy using a validation cohort. We implemented three machine learning algorithms to dichotomize the subjects. Then, we derived classification models from the training cohort using machine learning algorithms and implemented these models to predict OS and TFS in a validation cohort to assess efficacy. We developed and validated the prediction models based on the Transparent Reporting of a multivariable prediction model for Individual Prognosis or Diagnosis (TRIPOD) Statement. TRIPOD is intended to help readers better understand the study design, conduct, analysis, and interpretation of data and assess the validity, transportability, and application of study results^23,24^. All patients’ parents or legal guardians provided informed consent according to the Declaration of Helsinki. Institutional ethics committees approved the storage and collection of patient specimens. The ethics committee of the Nagoya University Graduate School of Medicine approved this study.

### Data sources

We used data from recently published international consensus definitions for the training cohort^19^. We also used data from 72 patients from Japan and the United States with available clinical information, which had not previously undergone methylation analysis for the validation cohort to examine the validity of the clinical model to predict OS and TFS.

### Statistical analysis

We compared the mutation frequency and other clinical features between the disease groups using Fisher’s exact test. OS and TFS were calculated using the Kaplan–Meier method. For the TFS analysis, transplantation and death from any cause were censored as events.

We implemented three different supervised machine learning algorithms (decision tree, SVM, and naïve Bayes model) to differentiate the methylation profiles. Features were extracted as older age (> 2 years old), male sex, decreased platelet count (< 33,000), elevated age-adjusted HbF, monosomy 7, and mutations in *PTPN11*, *CBL*, *NRAS*, and *KRAS* and were dichotomized (as in Supplementary Data)*.* Standard methods (fitcsvm, fitcnb, fitctree, and predict) from the Matlab^®^2020b Statistics and Machine Learning Toolbox with default parameter settings were used to derive the naïve Bayes classifier, the SVM-based classifier, and the decision tree. Tenfold cross-validation was employed to internally validate the models. First, we dichotomized the methylation profiles into two groups: HM/IM and LM. Second, we implemented each model to differentiate the methylation profiles into three categories: HM, IM, and LM. We first developed the decision tree algorithm as our primary analysis since it is an intuitive approach. Survival differences were tested using the log-rank test. All statistical analyses were performed using Stata 17.0 MP software (Stata Corp., College Station, TX, USA), and the machine learning algorithms were developed using MATLAB software (The MathWorks, Inc. Natick, MA, USA).

## Supplementary Information

Supplementary Figures.

Supplementary Information.

## Data Availability

The data underlying this article cannot be shared publicly to protect the study participants’ privacy.
