# Supplementary material for: Clinical parameter-based prediction of DNA methylation classification generates a prediction model of prognosis in patients with juvenile myelomonocytic leukemia
Source: Sci Rep. 2022 Aug 30;12:14753. doi: 10.1038/s41598-022-18733-4 (PMC9427938; doi:10.1038/s41598-022-18733-4)
Supplement: Supplementary file 1 — Supplementary Figures. [file 41598_2022_18733_MOESM1_ESM.docx]

**Clinical Parameter-Based Prediction of DNA Methylation Classification Generates a Prediction Model of Prognosis in Patients with Juvenile Myelomonocytic Leukemia**

Running title: Prediction of Methylation and Clinical Prediction in JMML

Takahiro Imaizumi^1^, Julia Meyer^2^, Manabu Wakamatsu^3^, Hironobu Kitazawa^3^, Norihiro Murakami^3^, Yusuke Okuno^4^, Taro Yoshida^3^, Daichi Sajiki^3^, Asahito Hama^5^, Seiji Kojima^3^, Yoshiyuki Takahashi^3^, Mignon Loh^2^, Elliot Stieglitz^2^, and Hideki Muramatsu*^3^

^1^Department of Advanced Medicine, Nagoya University Hospital, Nagoya, Japan

^2^Department of Pediatrics, Benioff Children’s Hospital, University of California, San Francisco, San Francisco, USA

^3^Department of Pediatrics, Nagoya University Graduate School of Medicine, Nagoya, Japan,

^4^Department of Virology, Nagoya City University Graduate School of Medical Sciences, Nagoya, Japan

^5^Department of Hematology and Oncology, Children’s Medical Center, Japanese Red Cross Nagoya First Hospital, Nagoya, Japan.

*Corresponding author.

**Supplementary Information**

**Figures**

**Supplementary Figure S1.** Overall survival and transplantation-free survival based on dichotomized DNA methylation classification using the SVM

**Supplementary Figure S2.** Overall survival and transplantation-free survival based on dichotomized DNA methylation classification using the Naïve Bayes model

**Supplementary Figure S3.** Overall survival and transplantation-free survival based on clinically predicted trichotomized DNA methylation using SVM

**Data**

**Supplementary Data** DNA Methylation classification and binary clinical parameters

**Supplementary Figure S1. Overall survival and transplantation-free survival based on dichotomized DNA methylation classification using the SVM**


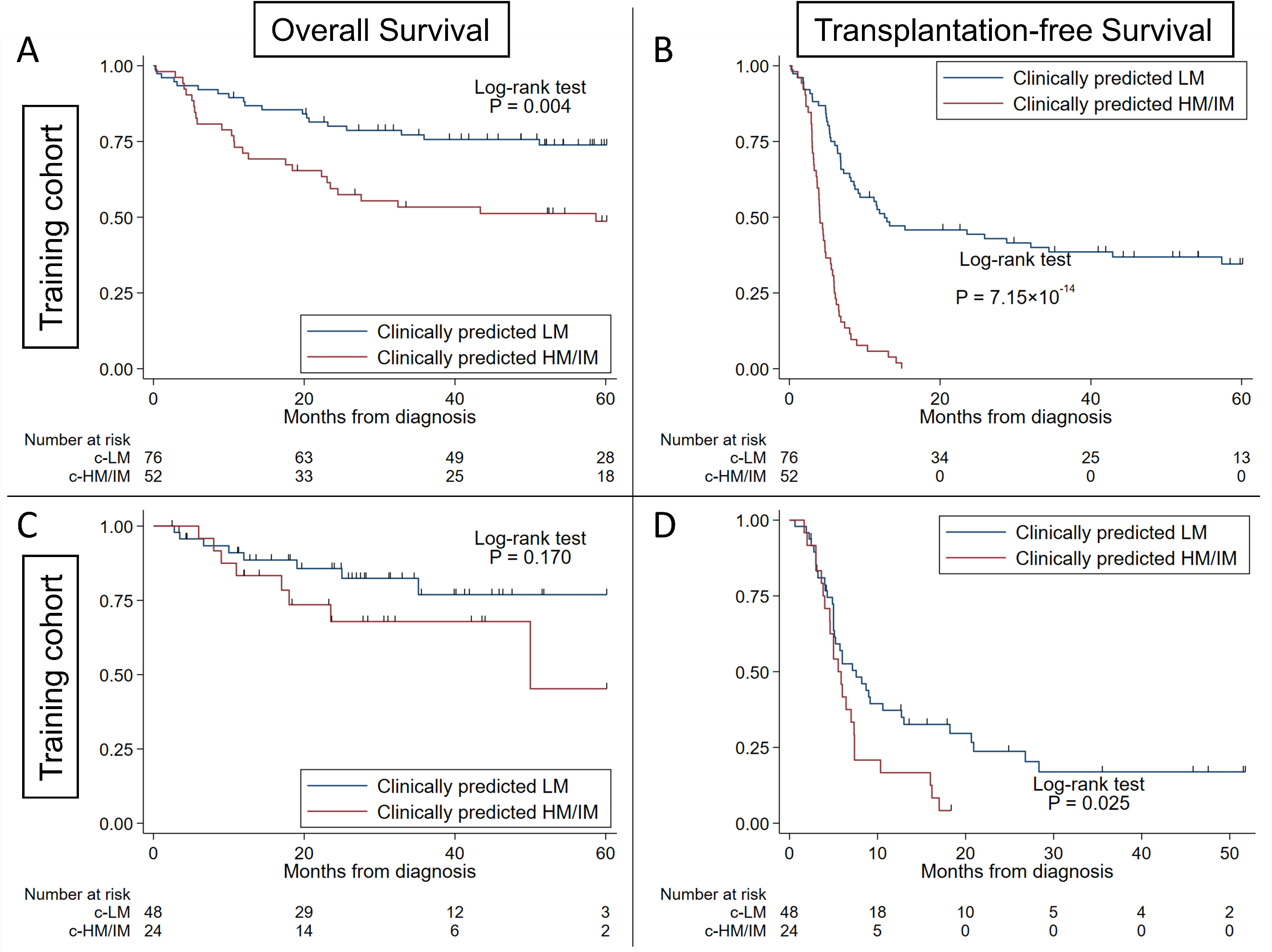


**A.** OS by clinically predicted methylation classification in the training cohort. **B.** TFS by clinically predicted methylation classification in the training cohort. **C.** OS in the validation cohort. **D.** TFS in the validation cohort. Survival functions were estimated using the Kaplan–Meier method, and statistical tests were performed using the log-rank test. Clinically predicted HM/IM or LM was significantly associated with both OS and TFS in the training cohort (P = 0.004 and 7.15 × 10^−14^, respectively), and associated with TFS in the validation cohort (P = 0.025). OS, overall survival; TFS, transplantation-free survival.

**Supplementary Figure S2. Overall survival and transplantation-free survival based on dichotomized DNA methylation classification using the Naïve Bayes model**


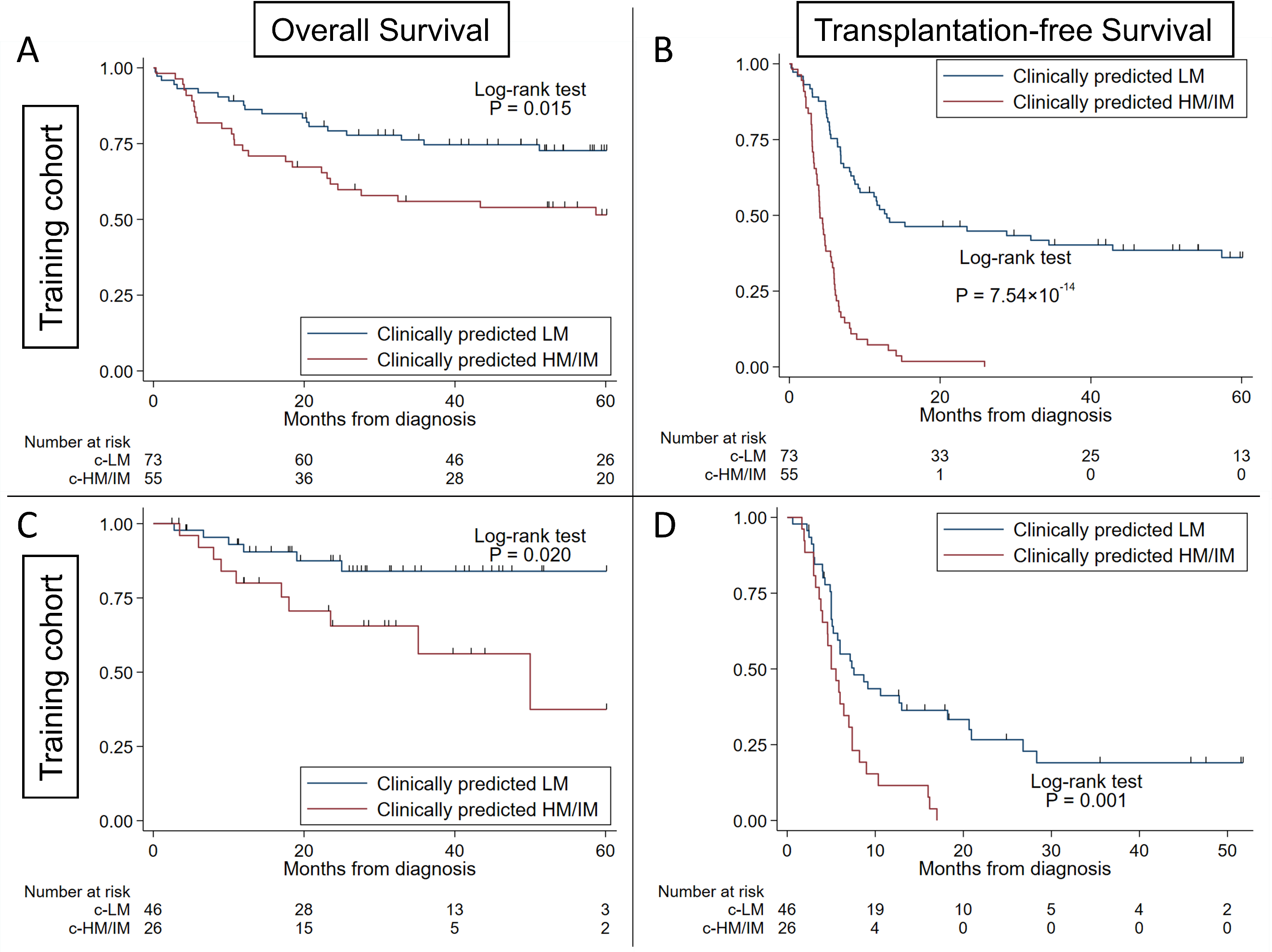


**A.** OS by clinically predicted methylation classification in the training cohort. **B.** TFS by clinically predicted methylation classification in the training cohort. **C.** OS in the validation cohort. **D.** TFS in the validation cohort. Survival functions were estimated using the Kaplan–Meier method, and statistical tests were performed using the log-rank test. Clinically predicted HM/IM or LM was significantly associated with both OS and TFS in the training cohort (P = 0.015 and 7.54 × 10^−14^, respectively) and associated with both OS and TFS in the validation cohort (P = 0.020 and 0.001, respectively). OS, overall survival; TFS, transplantation-free survival.

**Supplementary Figure S3. Overall survival and transplantation-free survival based on clinically predicted trichotomized DNA methylation using SVM**


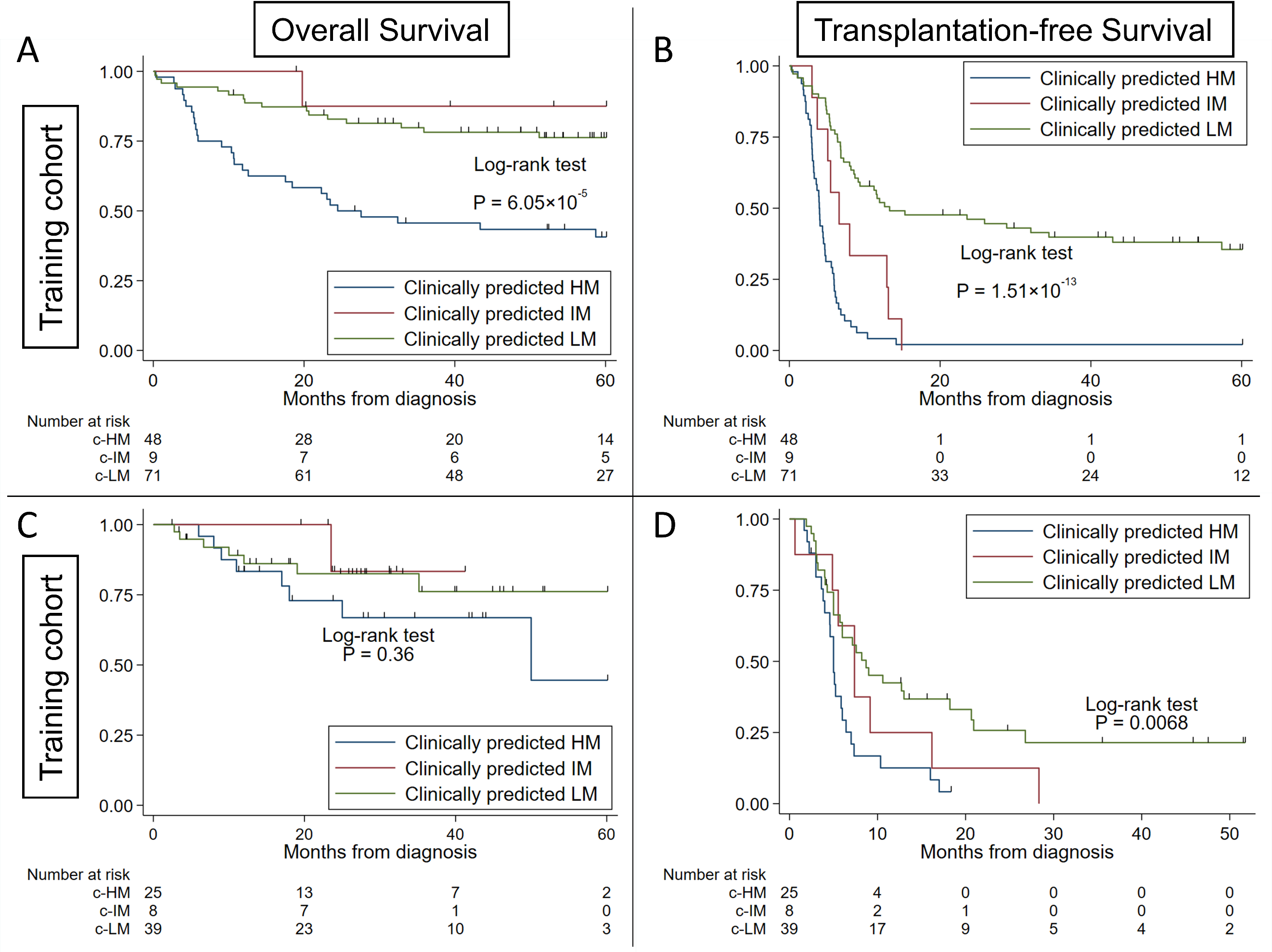


**A.** OS by clinically predicted methylation classification in the training cohort. **B.** TFS by clinically predicted methylation classification in the training cohort. **C.** OS in the validation cohort. **D.** TFS in the validation cohort. Survival functions were estimated using the Kaplan–Meier method, and statistical tests were performed using the log-rank test. Clinically predicted HM/IM or LM was significantly associated with both OS and TFS in the training cohort (P = 6.05 × 10^−5^ and 1.51 × 10^−13^, respectively) and associated with TFS in the validation cohort (P = 0.0068, respectively). OS, overall survival; TFS, transplantation-free survival.
